# A Mechanism for Adding the First Link in a Nascent Actin Filament Chain

**DOI:** 10.1371/journal.pbio.0020103

**Published:** 2004-04-13

The capacity for self-generated movement is a defining characteristic of animal life. With the molecular components of cellular locomotion conserved in organisms from protozoa to vertebrates, directed cell motility appears to be an ancient cell process, likely dating back a billion years. Most directed motion relies on the assembly, or polymerization, of actin proteins into filaments. Actin is one of the most abundant proteins in cells; about half of the cellular concentration of actin is bound together in filaments at any given time while the other half floats freely as “monomers” in the cytoplasm. The erection and demolition of actin filaments directs the cell motility that lays down the remarkable million miles of nerve cells that form the nervous system and drives a variety of fundamental biological processes, from effective immune response to embryonic development. Mutations in proteins that regulate actin assembly can lead to the abnormal cell migration associated with metastatic cancer. The actin cytoskeleton also provides the structural support for animal cells that the cell wall provides for plants.[Fig pbio-0020103-g001]

**Figure pbio-0020103-g001:**
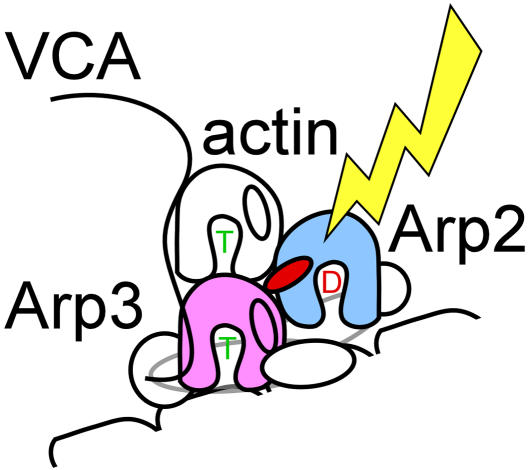
Actin addition

The molecular mechanisms underlying actin assembly and cell motility remained obscure until 1994, when Thomas Pollard and his colleagues discovered the protein complex that initiates actin polymerization. Called actin-related protein 2/3 (Arp2/3) complex, this molecular machine consists of seven subunits, including the two actin-related proteins. Free actin monomers are primed for rapid polymerization, but polymerization must be initiated by the Arp2/3 complex in a process referred to as nucleation. To nucleate a new filament, the Arp2/3 complex must be activated, a job accomplished by a family of proteins called WASP (after Wiskott Aldrich Syndrome, a genetic disease characterized by defects in platelet development and lymphocyte function). WASP proteins bind to both the Arp2/3 complex and an actin monomer. The Arp2/3 complex also binds two molecules of adenosine triphosphate (ATP) on the Arp2 and Arp3 subunits. ATP releases energy in a process called hydrolysis, which drives most energy-dependent processes, from actin polymerization to muscle contraction. The precise mechanisms governing Arp2/3 activation and nucleation are not known. Now Mark Dayel and Dyche Mullins show where hydrolysis occurs during this crucial first step in polymerization and use this finding to investigate the mechanisms that drive nucleation.

In previous experiments, Dayel and Mullins found that Arp2/3 appears to require hydrolysable ATP to effect nucleation. To determine when and if ATP hydrolysis occurs on the Arp2/3 complex, Dayel and Mullins developed a technique that allowed them to analyze the Arp2 and Arp3 subunits separately. Dayel and Mullins discovered that hydrolysis occurs only on the Arp2 subunit of the complex and that it happens during the step when WASP initiates the nucleation of a new filament. The researchers then used ATP hydrolysis on Arp2 to dissect the mechanism by which WASP activates the Arp2/3 complex and develop a model of nucleation. (All previous techniques required actin polymerization to monitor the activity of the Arp2/3 complex, but this technique offers a way to decouple activation from polymerization.) They find that WASP proteins activate the Arp2/3 complex by coordinating its interaction with an actin monomer—the first monomer of the new filament.

By developing a novel technique to monitor activation of the Arp2/3 complex, the authors contribute a new tool for further investigations of this central part of the cellular motility machinery. And by showing how Arp2/3 is activated, they offer important insights into the workings of a multiprotein cellular machine and the mechanisms that cells enlist to control their shape and motility—which could suggest potential drug targets to inhibit the abnormal cell movement characteristic of cancer and other diseases.

